# Supplementary material for: Investigating Mycobacterium tuberculosis sufR (rv1460) in vitro and ex vivo expression and immunogenicity
Source: PLoS One. 2023 Jun 15;18(6):e0286965. doi: 10.1371/journal.pone.0286965 (PMC10270350; doi:10.1371/journal.pone.0286965)
Supplement: S1 File — (DOCX) [file pone.0286965.s001.docx]

**Investigating *Mycobacterium tuberculosis* sufR (Rv1460) *in vitro* and *ex vivo* expression and immunogenicity:** Supplementary File

| **Plasmids / Strains** | **Description** | **Source** |
| --- | --- | --- |
| **Plasmids**  pJET1.2 | Linearized blunt end cloning vector (Amp^R^) | CloneJet |
| pJET1.2SufR-123mCherry | pJET1.2 vector containing DNA fusion fragment SufR _123mCherry. | This study |
| pMV306 | Mycobacterial integrating shuttle vector with an hygromycin resistance gene and an integrase gene which allows the vector to integrate into the *attB*site of the mycobacterial genome. | (Stover *et al.*, 1991) |
| pMV306_123mCherry | pMV306 vector with 123 bp of *SufR* promotor cloned upstream of a promotorless *mcherry* gene. | This study |
| **Strains**  *E. coli* XL1 Blue | *recA1endA1gyrA96thi*-*1hsdR17supE44relA1lac*[F ́ *proABlacI*^q^*Z∆M15* Tn*10*] (Tet^R^) | Stratagene |
| H37Rv | H37RvMA (ATCC: 27294) | Ioerger*et al.*, 2010 |
| H37Rv *attB*::pMV306 | Derivative of H37RV containing pMV306 mycobacterial integrating vector. | This study |
| H37Rv *attB*::pMV306_123mCherry | Derivative of H37RV containing pMV306_123mCherry mycobacterial integrating vector. | This study |
| *M. smegmatis* mc^2^155 with pSTCHARGE3 plasmid | hsp60(ribo)-turboFP635 (inducible TurboFP635 under control of theophylline-inducible riboswitch), Kan^R^, episomal | Mouton *et al* (2016) |

**S1 Table. Plasmids and strains used in this study**

**S2 Table. List of primers used for the construction of fluorescent reporter vector**

| **Primer name** | **Sequence (5’ to 3’)** | **Binding position** | **Product size** |
| --- | --- | --- | --- |
| SufRPf | GATATCGCCATTGGTGCAGCCTAAC (EcoRv) | Binds 123 bp upstream of *sufR* start site. | 123 bp |
| SufRPr | AACATCAGTGTGACAAAATTCCGTTG | Binds 2 bp upstream of *sufR* start site |  |
| pCherF | ATGGCGATCATCAAGGAGTT | Binds *mCherry* at the start codon. | 730 bp |
| pCherR | GACGGTATCGATAAGCTTTCAC (Hind III) | Binds 2 bp from the *mCherry* stop codon. |  |
| Ovlp | AATTTTGTCACACTGATGTTATGGCGATCATCAAG | Contains 20bp of 3’ end of *sufR* promotor region fused to 15 bp of 5’ end of *mCherry* gene 20.  Ovlp plus SufRPf plus pCherR | 853 bp |
| List of primers used for cDNA synthesis and qPCR | | | |
| SufRRT | TCATCGGGACGCTCCTTCGG | Binds 788 bp upstream from *SufR* start site | N/A |
| SufRPf | GAATTGTGCGAAACCGACGAGCAGCAG | Binds 640 bp from *sufR* start site | 106 bp |
| SufRPr | CAGGGGTACGTGGGTGGTG | Binds 732 bp from *sufR* start site |  |
| mCherryRTP | TGCTTGATCTCGCCCTTCA | 503 bp downstream from *mCherry* start site | N/A |
| pCherF | GACATCCCGGACTACCTGAA | Binds *mCherry* 139 bp downstream of the start codon. | 194 bp |
| pCherR | AGCCCATGGTCTTCTTCTGCAT | Binds 333 bp from the *mCherry* start codon. |  |
| SigART | CTGACATGGGGGCCCGCTACGTTG | Binds 19 bp downstream of *SigA* | N/A |
| SigAF | TGCAGTCGGTGCTGGACAC | Binds 1379 bp from start of *SigA* | 195 bp |
| SigAR | CGCGCAGGACCTGTGAGCGG | Bins 1552 bp from start of *SigA* |  |

RE sites are underlined and the name of the enzyme is listed in brackets

| **Antibody** | **Fluorophore** | **Company** |
| --- | --- | --- |
| CD3 | PerCP | Biocom Africa |
| CD4 | APC/H7 | BD Bioscience |
| CD8 | BV510 | Biocom Africa |
| CD19 | FITC | Biocom Africa |
| IFN-g | BV421 | Biocom Africa |
| TNF-a | PE | Biocom Africa |
| IL-2 | PE/Cy7 | Biocom Africa |
| IL-10 | APC | Biocom Africa |

**S3 Table. Antibodies used for flow cytometry**

**S4 Table.** **Clinical and demographical characteristics of study participants**

|  | Active TB (*n*=20) | QFN pos (*n*=9) | QFN neg (*n*=17) |
| --- | --- | --- | --- |
| Male, *n* (%) | 11 (55) | 4 (44.4) | 11 (64,7) |
| Female, *n* (%) | 9 (45) | 5 (55.6) | 6 (35,2) |
| Mean Age, *n* (%) | 20 (37.7) | 9 (42.4) | 17 (30.6) |

Boolean gating:

CD19 + CD8+

IL-2 IL-2

IL-10 IL-10

IFN-g IFN-g

TNF-a TNF-a


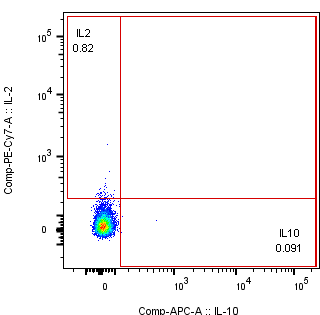

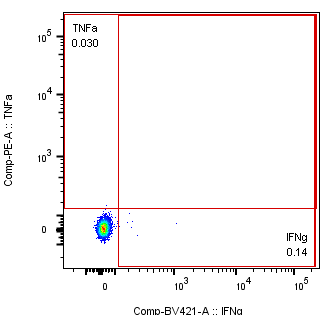

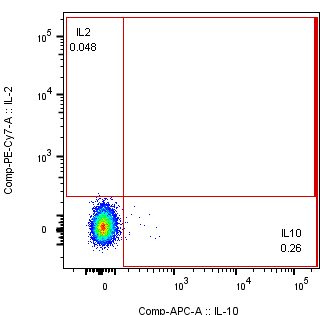

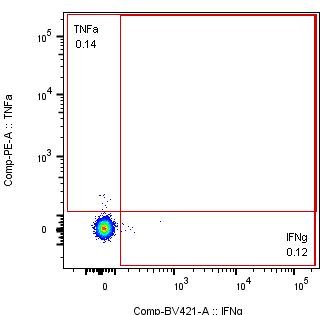


E 1

TNF-a

IL-2

IL-2

TNF-a

IFN-g

IFN-g

IL-10

IL-10

C 1


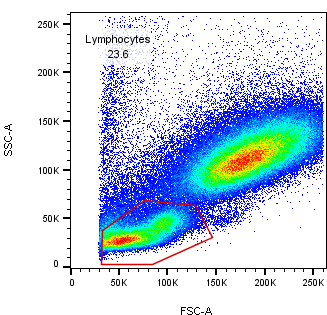

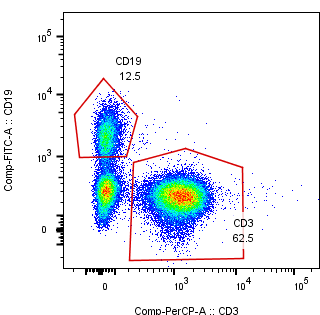

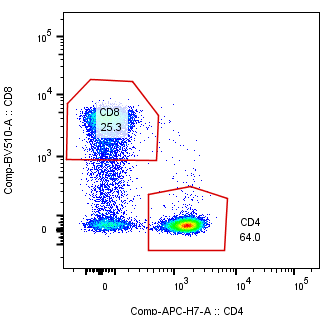


B

Boolean gating:

CD4+

IL-10

IL-2

IFN-g

TNF-a

E

D

C

A

D 1

CD19

CD8

CD3

CD4


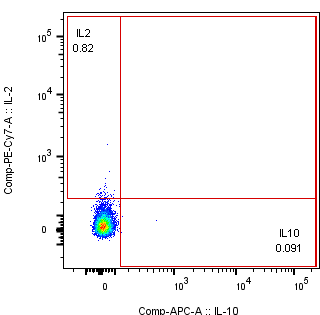

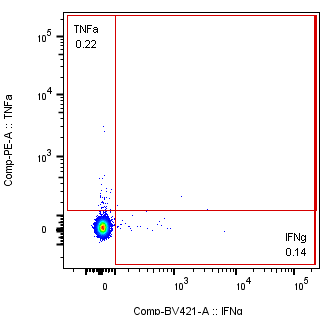


IFN-g

IL-10

IL-2

TNF-a

**S1 Figure. Gating strategy for analysis of phenotype screening of T and B cells.** (A) Lymphocytes were gated on by plotting SSC-A vs FSC-A, followed by gating (B) CD3+ T cells and (C) CD19+ B cells (CD3-). T cells were further subdivided in (D) CD4+ and CD8+ T cells. Boolean gating strategy (C1, E1 and D1) was used to determine the frequencies of each potential combination cytokine produced by CD4+, CD8+ and CD19+.

In the CD8+ subset there was no significant difference between active TB, QFN pos and QFN neg groups following 12 hours of being unstimulated, SufR and BCG stimulated. Following 12-hour PHA stimulation significantly higher frequencies of CD8+IFN-g+ (*p* < 0.01), CD8+IL-2+ (*p* = 0.05) and CD8+TNF-a+ (*p* < 0.01) was seen in the QFN neg group (*p* < 0.01; *p* = 0.05 and *p* < 0.01) vs active TB after 12-hour PHA stimulation (S2 Fig).

Significantly higher frequencies of CD8+IL-10+ (*p* < 0.01) and CD8+TNF-a+ (*p* = 0.01) were observed in the QFN pos respectively vs QFN neg following 7-day PHA stimulation (Supplementary Figure 1). There was however no significant difference in the frequency of CD8+IFN-g+ and CD8+IL-2+ between active TB, QFN pos and QFN neg groups following 7-day SufR, BCG and PHA stimulation. We observed significantly higher frequencies for CD8+IFN-g+ and CD8+TNF-a+ in the active TB, QFN pos and QFN neg group following 7-day SufR stimulation compared to the unstimulated condition (S2 Fig).

Frequencies (not significant) of the CD8+IFNg+IL2+IL10-TNFa- multifunctional T cell subset in the active TB (*p* = 0.09) compared to the QFN pos group following 7-day SufR stimulation. Increased frequencies of CD8+IFNg+IL2-IL10-TNFa+, CD8+IFNg+IL2-IL10-TNFa-, CD8+IFNg-IL2-IL10-TNFa+ multifunctional T cells were measured in all three groups after 7-day SufR stimulation when compared to the unstimulated condition (S5 Table).

Significantly higher frequencies were observed in the multifunction T cell subset CD8+IFNg+IL2-IL10+TNFa+ in QFN neg group compared to active TB (*p* = 0.03) and QFN pos group (*p* = 0.01) following 7-day PHA stimulation. Also, significantly higher frequencies were observed in the multifunction T cell subset CD8+IFNg-IL2-IL10+TNFa+ in the QFN pos group vs active TB (p = 0.00) and QFN neg group (*p* = 0.01) and in the QFN neg group vs active TB (*p* = 0.02) following 7-day PHA stimulation. Overall, we observed significantly higher frequencies of the multifunctional T cells following 7-day stimulation compared to 12-hour stimulation (S5 Table).

B

A

D

C

**S2 Figure.** Flow cytometry phenotype screening of CD8+ T cell subset in whole blood from active TB group (n=20), QFN pos group (n=9) and QFN neg group (n=17) after 12 hours and 7 days of being unstimulated and stimulated with SufR, BCG and PHA. We measured the frequency of CD8+ T cell subsets (A) CD8+IFN-g, (B) CD8+IL-2+, (C) CD8+IL-10+ and (D) CD8TNF-a+ cells. ANOVA test was performed to determine statistically significant differences between groups. ANOVA test was performed to determine statistically significant differences between groups. Vertical bars denote 0.95 CI.

**S5 Table. Multifunctional CD8+T cell subset frequencies of in active TB, QuantiFERON negative and QuantiFERON positive groups following BCG and PHA stimulation at 2 timepoints.**

|  |  | **12-hour** | | | **7-day** | | |
| --- | --- | --- | --- | --- | --- | --- | --- |
|  |  | Active TB VS  QFN pos | Active TB VS  QFN neg | QFN neg VS  QFN pos | Active TB VS  QFN pos | Active TB VS  QFN neg | QFN neg VS  QFN pos |
| **CD8+IFNg+IL2+IL10+TNFa+** | BCG | ns | ns | ns | ns | ns | ns |
|  | PHA | ns | ns | ns | ns | ns | ns |
| **CD8+IFNg+IL2+IL10-TNFa-** | BCG | ns | ns | ns | ns | ns | ns |
|  | PHA | ns | ns | ns | ns | ns | ns |
| **CD8+IFNg+IL2-IL10+TNFa+** | BCG | ns | ns | ns | ns | ns | ns |
|  | PHA | ns | ns | ns | ns | *p* = 0.03 | *p* = 0.01 |
| **CD8+IFNg+IL2-IL10+TNFa-** | BCG | ns | ns | ns | *p* = 0.03 | ns | ns |
|  | PHA | ns | ns | ns | ns | ns | ns |
| **CD8+IFNg+IL2-IL10-TNFa+** | BCG | ns | *p* = 0.03 | ns | ns | ns | ns |
|  | PHA | ns | *p* = 0.04 | ns | ns | *p* = 0.04 | ns |
| **CD8+IFNg-IL2+IL10-TNFa-** | BCG | ns | ns | ns | ns | ns | ns |
|  | PHA | ns | *p* = 0.01 | ns | ns | ns | ns |
| **CD8+IFNg-IL2+IL10-TNFa-** | BCG | ns | ns | ns | ns | ns | ns |
|  | PHA | ns | *p* = 0.01 | ns | ns | ns | ns |
| **CD8+IFNg-IL2+IL10-TNFa+** | BCG | ns | ns | ns | ns | ns | ns |
|  | PHA | ns | *p* = 0.01 | ns | *p* = 0.03 | ns | ns |
| **CD8+IFNg-IL2+IL10-TNFa-** | BCG | ns | ns | ns | ns | ns | ns |
|  | PHA | ns | ns | ns | ns | ns | ns |
| **CD8+IFNg-IL2-IL10+TNFa+** | BCG | ns | ns | ns | ns | *p* = 0.01 | ns |
|  | PHA | ns | ns | ns | *p* = 0.00 | *p* = 0.02 | *p* = 0.01 |
| **CD8+IFNg-IL2-IL10+TNFa-** | BCG | ns | ns | ns | ns | ns | ns |
|  | PHA | ns | ns | ns | ns | ns | ns |
| **CD8+IFNg-IL2-IL10-TNFa+** | BCG | ns | ns | ns | ns | *p* = 0.02 | ns |
|  | PHA | ns | ns | ns | ns | ns | ns |
| **CD8+IFNg-IL2-IL10-TNFa-** | BCG | ns | ns | ns | ns | ns | ns |
|  | PHA | ns | *p* = 0.00 | ns | ns | ns | *p* = 0.03 |

No significant difference in the frequency of CD19+IFN-g+ and CD19+IL-10+ cells in the active TB, QFN pos and QFN neg groups following 12-hour stimulation with SufR, BCG and PHA. Higher frequencies of CD19+IL-2+ although not significant was measured in the QFN pos group (*p* = 0.07) when compared to active TB after 12-hour SufR stimulation. There were also significantly higher levels of CD19+TNF-a+ measured in QFN neg group (*p* < 0.01) when compared to active TB group following 12-hour PHA stimulation (S3 Fig).

No significant differences were observed in the CD19+IL-2+ levels in the three groups following 7-day SufR, PHA stimulation and the unstimulated conditions. Significantly higher levels of CD19+IFN-g+ cells in the QFN neg group when compared active TB (*p* = 0.03) and QFN pos (*p* = 0.02) following 7-day BCG stimulation. Significantly higher frequency of CD19+IL-10+ in the active TB group when compared to QFN pos (*p* = 0.01) and QFN neg (*p* = 0.01) following 7-day BCG stimulation. Inversely, significantly lower frequencies of CD19+TNF-a+ in the active TB group when compared to QFN pos (*p* = 0.02) and QFN neg (*p* = 0.03) following 7-day PHA stimulation (S3 Fig).

A

B

D

C

 ****

**S3 Figure 3.** Flow cytometry phenotype screening of CD19+ B cell subset in whole blood from active TB group (n = 20), QFN pos group (n = 9) and QFN neg group (n = 17) after 12 hours and 7 days of being unstimulated (US) and stimulated with SufR, BCG and PHA. The CD19+ B cell subsets (A) CD19+IFN-g, (B) CD19+IL-2+, (C) CD19+IL-10+ and (D) CD19+TNF-a+. ANOVA test was performed to determine statistically significant differences between groups. ANOVA test was performed to determine statistically significant differences between groups. Vertical bars denote 0.95 CI.

**S6 Table. CD19+ B cell subset frequencies of in active TB, QuantiFERON negative and QuantiFERON positive groups following SufR, BCG and PHA stimulation at 2 time points.**

|  |  | **12-hour** | | | **7-day** | | |
| --- | --- | --- | --- | --- | --- | --- | --- |
|  |  | Active TB VS  QFN pos | ActiveTB VS  QFN neg | QFN neg VS  QFN pos | ActiveTB VS  QFN pos | ActiveTB VS  QFN neg | QFN neg VS  QFN pos |
| **CD19+IFNg+IL2-IL10+**  **TNFa+** | SufR | ns | ns | ns | ns | ns | ns |
|  | BCG | ns | ns | ns | ns | *p* = 0.01 | ns |
|  | PHA | ns | ns | ns | ns | ns | ns |
| **CD19+IFNg+IL2-IL10+**  **TNFa-** | SufR | ns | ns | ns | ns | ns | ns |
|  | BCG | ns | ns | ns | ns | ns | ns |
|  | PHA | ns | *p* = 0.05 | ns | ns | ns | ns |
| **CD19+IFNg+IL2-IL10-TNFa+** | SufR | ns | ns | ns | ns | ns | ns |
|  | BCG | ns | ns | ns | ns | *p* = 0.01 | *p* = 0.02 |
|  | PHA | ns | ns | ns | ns | ns | ns |
| **CD19+IFNg+IL2-IL10-TNFa-** | SufR | ns | ns | ns | ns | ns | ns |
|  | BCG | ns | ns | ns | ns | ns | *p* = 0.04 |
|  | PHA | ns | ns | ns | ns | ns | ns |
| **CD19+IFNg-IL2-+IL10-TNFa-** | SufR | *p* = 0.02 | ns | ns | ns | ns | ns |
|  | BCG | ns | ns | ns | ns | ns | ns |
|  | PHA | ns | ns | ns | ns | ns | ns |
| **CD19+IFNg-IL2+IL10-TNFa+** | SufR | ns | ns | ns | ns | ns | ns |
|  | BCG | ns | ns | ns | ns | ns | ns |
|  | PHA | ns | ns | ns | ns | ns | ns |
| **CD19+IFNg-IL2-IL10+ TNFa+** | SufR | ns | ns | ns | ns | ns | ns |
|  | BCG | ns | *p* = 0.01 | ns | ns | ns | ns |
|  | PHA | *p* = 0.03 | ns | ns | *p* = 0.03 | ns | ns |
| **CD19+IFNg-IL2-IL10+ TNFa-** | SufR | ns | ns | ns | ns | ns | ns |
|  | BCG | ns | ns | ns | *p* = 0.00 | *p* = 0.01 | ns |
|  | PHA | ns | ns | ns | ns | ns | ns |
| **CD19+IFNg-IL2-IL10- TNFa+** | SufR | ns | ns | ns | ns | *p* = 0.02 | ns |
|  | BCG | ns | ns | ns | ns | ns | ns |
|  | PHA | ns | *p* = 0.01 | ns | *p* = 0.02 | *p* = 0.04 | ns |
|  | SufR | ns | ns | ns | ns | ns | ns |
| **CD19+IFNg-IL2-IL10-TNFa-** | BCG | ns | ns | ns | ns | ns | ns |
|  | PHA | ns | *p* = 0.01 | ns | ns | ns | ns |
|  |  |  |  |  |  |  |  |
